# Supplementary material for: Bessel beam optical coherence microscopy enables multiscale assessment of cerebrovascular network morphology and function
Source: Light Sci Appl. 2024 Nov 11;13:307. doi: 10.1038/s41377-024-01649-1 (PMC11551179; doi:10.1038/s41377-024-01649-1)
Supplement: Supplementary file 1 — Supplementary Data and Discussion [file 41377_2024_1649_MOESM1_ESM.docx]

**Supplementary Information for:**Bessel Beam Optical Coherence Microscopy Enables Multiscale Assessment of Cerebrovascular Network Morphology and Function

Lukas Glandorf^1,2^, Bastian Wittmann^3^, Jeanne Droux^4^, Chaim Glück^1^, Bruno Weber^1^,
Susanne Wegener^4^, Mohamad El Amki^4^, Rainer Leitgeb^5^, Bjoern Menze^3^, Daniel Razansky^1,2,*^

*^1^Institute of Pharmacology and Toxicology & Institute for Biomedical Engineering, Faculty of Medicine, University of Zurich, Zurich, Switzerland*
*^2^Institute for Biomedical Engineering,* *Department of Information Technology and Electrical Engineering, ETH Zurich, Zurich, Switzerland*
*^3^Department of Quantitative Biomedicine, University of Zurich, Zurich, Switzerland*

*^4^ Department of Neurology, University Hospital Zurich and University of Zurich, Zurich, Switzerland*
*^5^Center for Medical Physics and Biomedical Engineering, Medical University Vienna, Vienna, Austria*
 [**daniel.razansky@uzh.ch*](mailto:*daniel.razansky@uzh.ch)

**
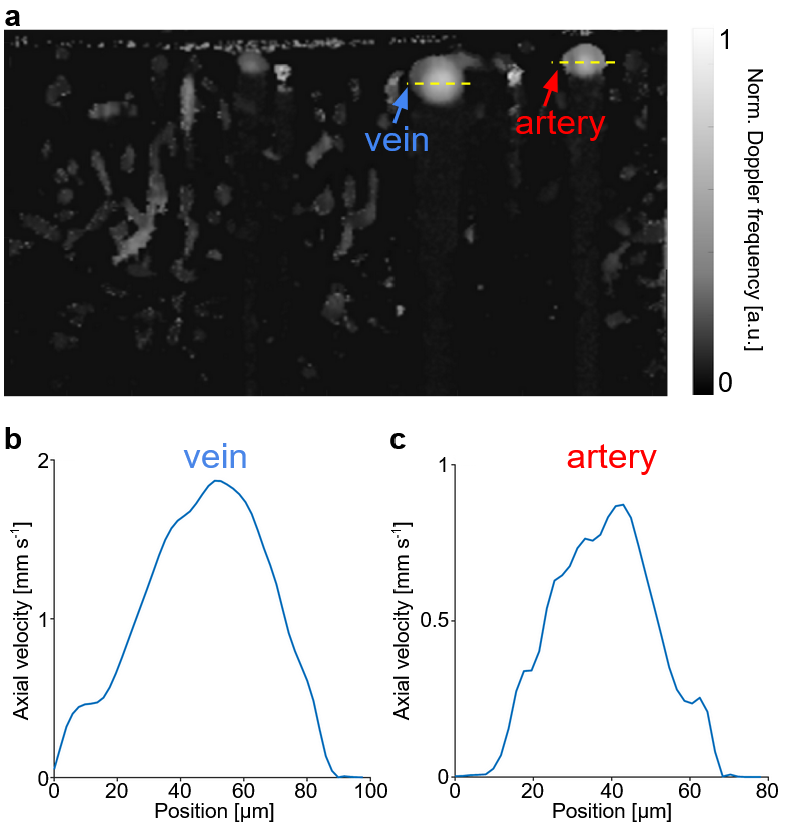
Supplementary Figure 1:**

Figure S1: Blood flow gradient within vessel cross-sections. a) Side view of a Doppler frequency volume. An artery and a vein are marked by red and blue arrows, respectively. The dashed yellow lines indicate the line profiles shown in b) and c). b) Axial velocity line profile within the vein, extracted along the yellow dashed line in a). c) Axial velocity line profile within the artery, extracted along the yellow dashed line in a). Both profiles in b) and c) exhibit approximately parabolic shape as expected in large vessels with laminar flow characteristics.


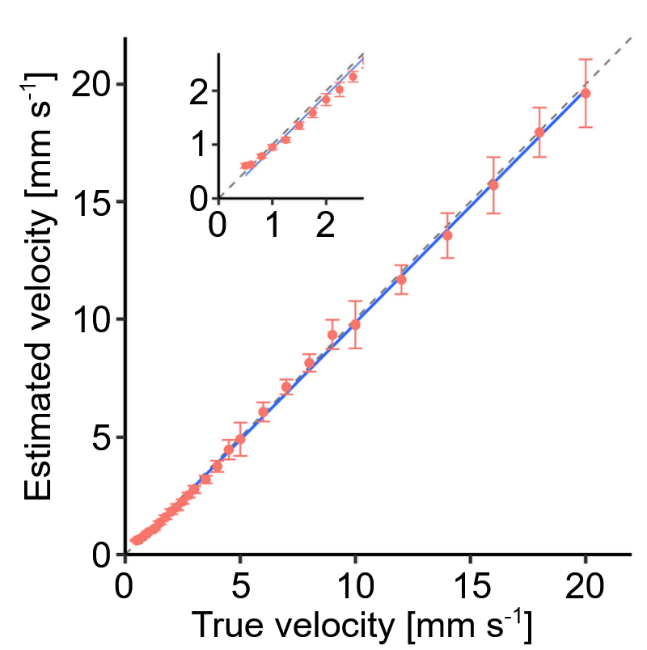
**Supplementary Figure 2:**

Figure S2: Phantom study of velocity measurement in 180 μm tubing and 1.5% intralipid solution. Good agreement between the ground truth and estimated flow velocities is found. Flow velocities above ≈20 mm/s result in Doppler aliasing due to the 8° angle of the tubing but do not constitute an upper limit for velocity estimation in-vivo. The pial vasculature, exhibiting the highest flow velocities, typically has angles closer to 0°, preventing aliasing.

**Supplementary Figure 3:**


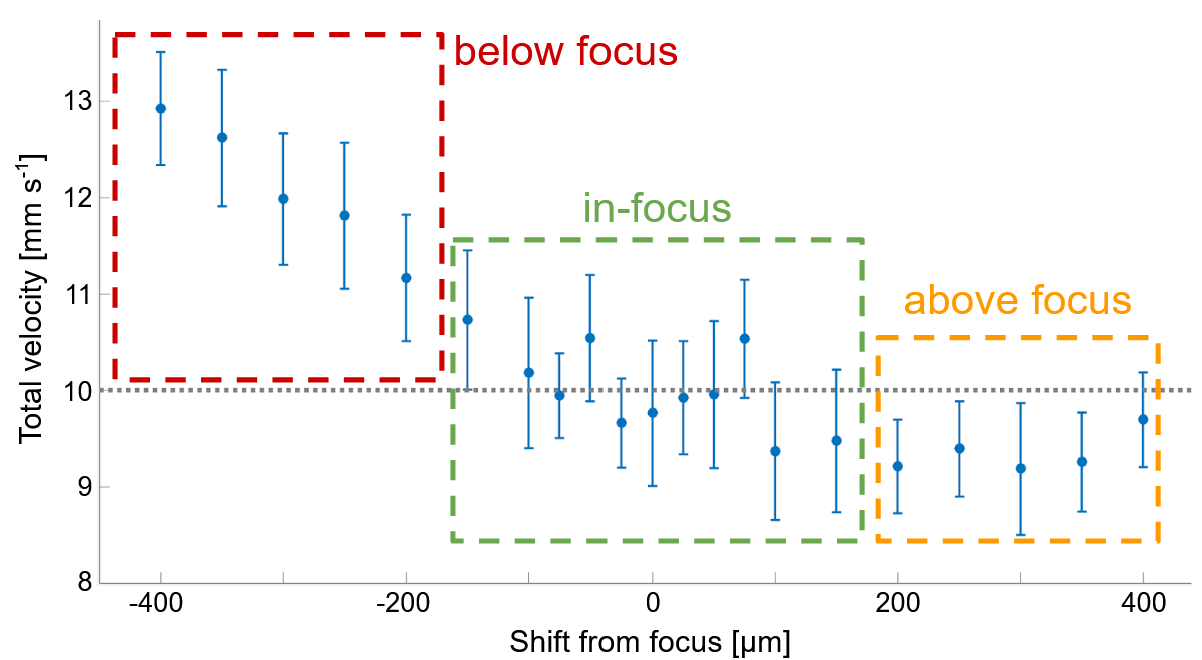


Figure S3: Phantom study of out-of-focus velocity estimation in 180 μm tubing and 1.5% intralipid solution. Flow well above the focus is slightly underestimated (<10%). Only flow far below the in-focus volume showed flow overestimation in excess of 10%. For in-vivo studies, this would not be considered because the microvasculature is not segmented in the regions far below the in-focus volume.


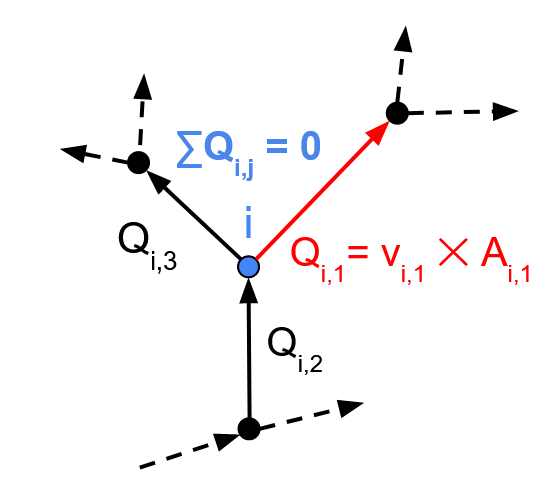
**Supplementary Figure 4:**

Figure S4: Estimating missing velocities through conservation of mass at branch points.


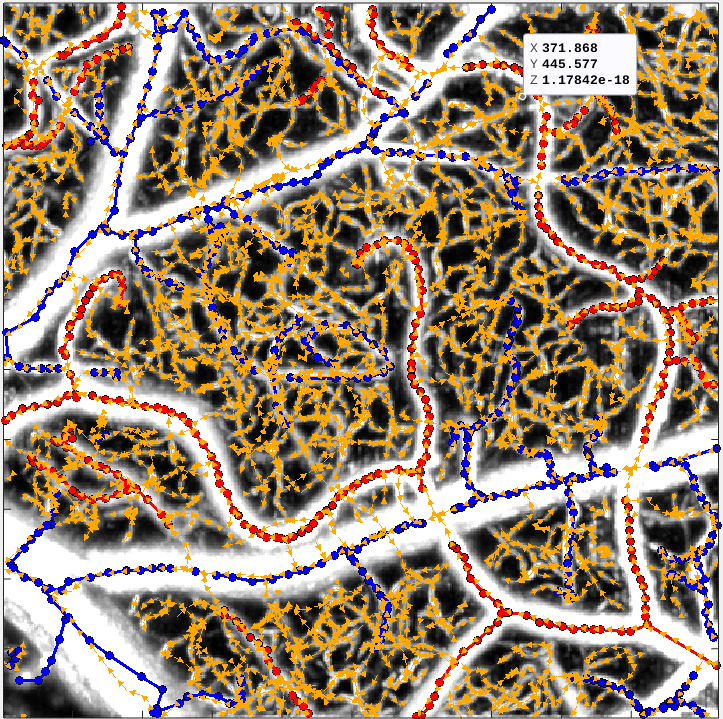
**Supplementary Figure 5:**

Figure S5: Angiogram MIP with overlayed flow directions (orange) and manual artery (red) and vein (blue) labels.

**arteries**

**veins**

**direction**

**
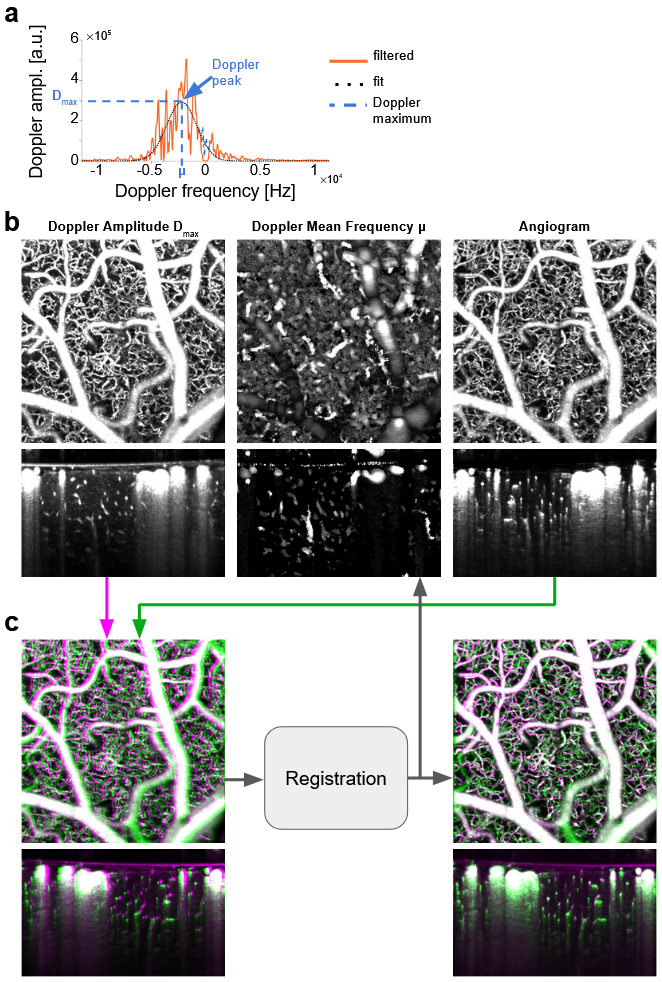
Supplementary Figure 6:**

Figure S6: Registration using Doppler amplitude. a) Doppler spectrum from one exemplary voxel. The Doppler amplitude D_max_ at the peak of the fitted modified Gaussian function is used to construct an OCTA-like angiogram. b) Comparison of Doppler amplitude angiogram (left), Doppler frequency μ (middle) and OCTA angiogram (right). The Doppler amplitude volumes are much closer in appearance to the OCTA angiogram than the quantitative Doppler frequency data. Therefore, Doppler amplitude is used for registration to the OCTA angiogram. c) Before and after overlay of the Doppler to OCTA registration. The acquired registration transform is then applied to the quantitative Doppler data.


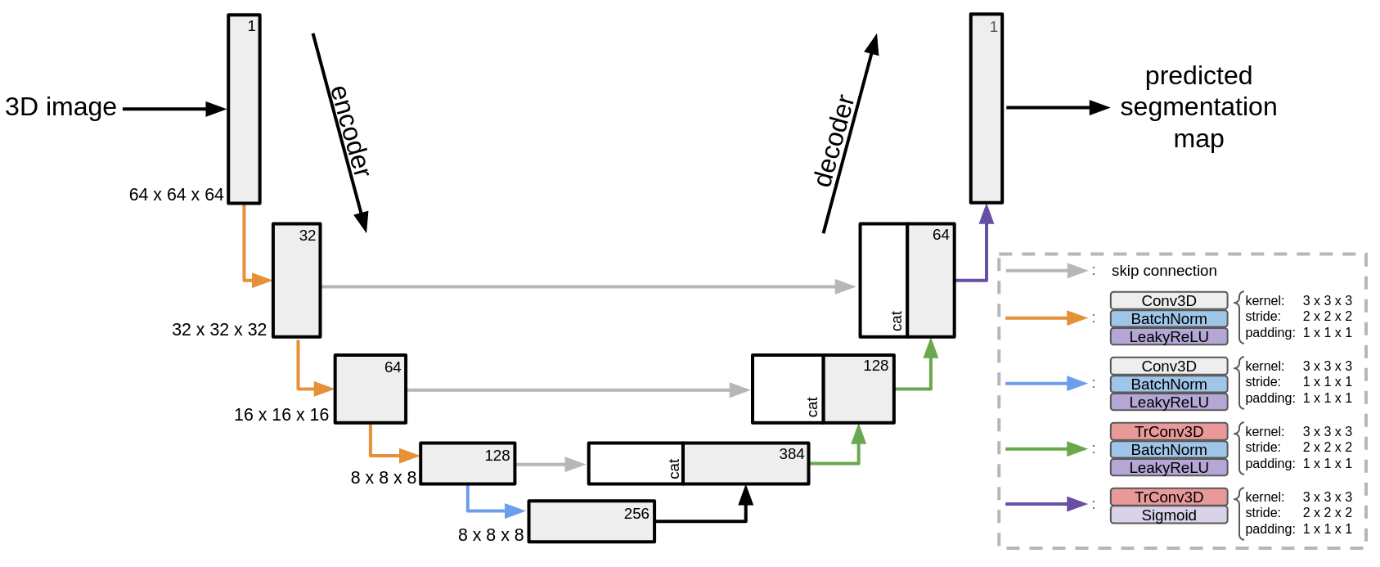
**Supplementary Figure 7:**

Figure S7: Detailed architecture of our employed 3D U-Net. To provide a concise and clear visualization, we represent feature maps as rectangles. In this context, the height of a rectangle reflects the spatial dimensions of feature maps (see left to feature maps) while its width indicates the number of channels (see right upper corner of feature maps). Please note that the color coding of arrows refers to different operations. We opted for the use of batch normalization layers and Leaky ReLU activation functions for stable, accelerated training with increased gradient flow. To scale the output of our U-Net to the desired range, we finally apply a Sigmoid function. The U-Net’s encoder utilizes strided 3D convolutions, whereas the decoder makes use of strided 3D transposed convolutions. The abbreviation ‘cat’ indicates the concatenation of two feature maps of the same spatial dimensions in the channel dimension. The entire 3D U-Net variant processes 1,939,681 trainable parameters.

**Supplementary Figure 8:**


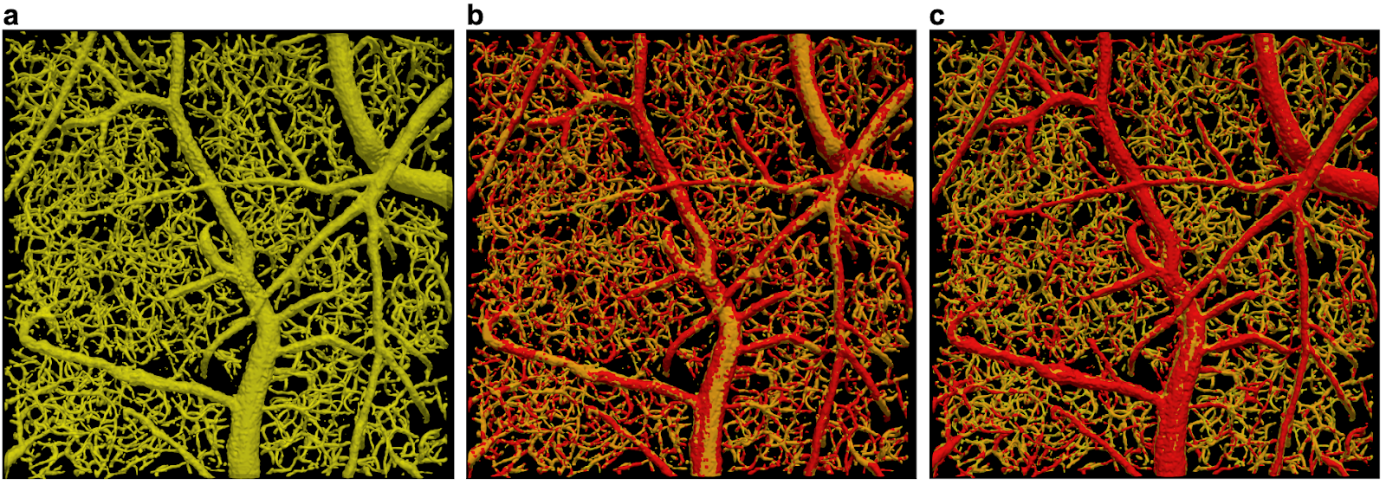


Figure S8: Qualitative comparison of segmentation maps. a) Segmentation map generated by the U-Net pre-trained on synthetic data and finetuned on manually annotated data (green). b) Segmentation map generated by the U-Net trained solely on manually annotated data (red) overlaid with (a). Analysis of (b) indicates that pre-training on synthetic data enables us to capture small capillaries more accurately. c) Segmentation map generated by the U-Net trained solely on synthetic data (red) overlaid with (a).

**
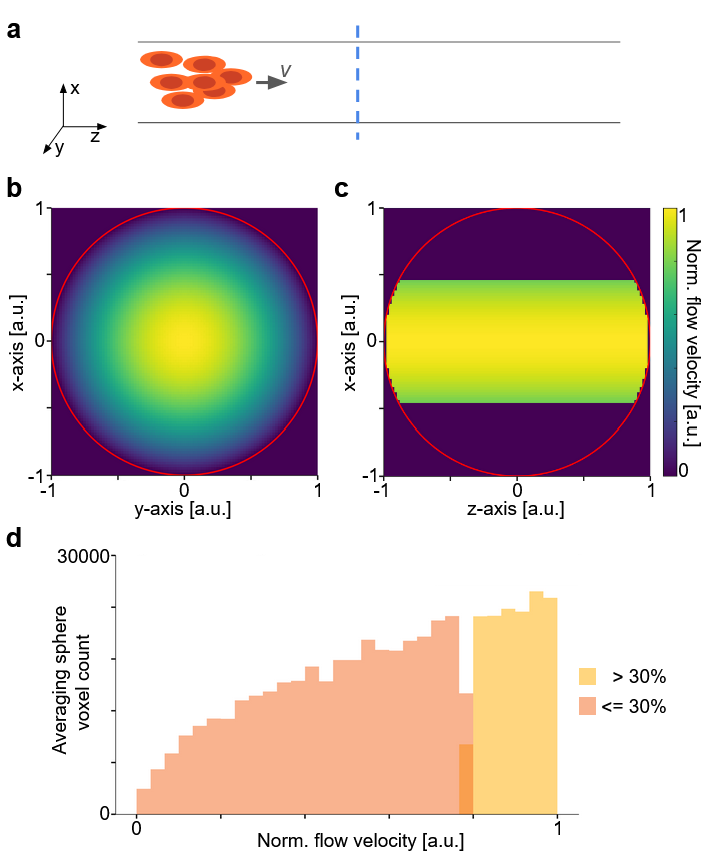
Supplementary Figure 9:**

Figure S9: Spherical averaging along elongated vessels. a) Exemplary blood vessel along the z-axis. b) Cross-sectional view of the simulated vessel in a) at the dashed blue line with a normalized radius of 1 that is considered to be much larger than the system resolution and exhibits laminar flow. The flow velocity is normalized to 1 at the center and follows a rotationally symmetric parabolic profile towards the edges. The red line marks the simulated vessel wall. b) Cross-sectional view of the averaging sphere. The flow profile from b) is repeated along z-axis to form an elongated simulated vessel with flow along the z-axis like in a). Values below 30 % of the maximum are set to zero in accordance to our averaging scheme in local spheres with radius equal to the vessel radius. c) Histogram of voxels within the averaging sphere. Bars in yellow indicated values above the 30 % threshold, values in red below. The cut-off is at approximately 79 % of the normalized maximum flow velocity.

**Supplementary Table 1:**

Table S1: Relevance of model training data. We compare three U-Net models of same configuration trained on either solely synthetic data (first row), solely manually annotated data (second row), and pre-trained on synthetic data and finetuned on manually annotated data (third row). The evaluation is done separately for the test set, previously unseen by the model, and the validation dataset, used for model development. We report Dice, centerline (cl-) Dice, and Accuracy values. Best performance is highlighted in bold. The model trained on both real and synthetic data, which is also the one used in our manuscript, performs best on both datasets and with respect to all metrics.

| *model* | *split* | *Dice* | *cl-Dice* | *Accuracy* |
| --- | --- | --- | --- | --- |
| *synthetic* | test | 0.5637 | 0.6085 | 0.9534 |
|  | val | 0.6110 | 0.6787 | 0.9718 |
| *manual_annotated* | test | 0.7737 | 0.7544 | 0.9718 |
|  | val | 0.7748 | 0.7713 | 0.9822 |
| *manual_annotated_synthetic* (model used in our manuscript) | test | **0.7891** | **0.7592** | **0.9739** |
|  | val | **0.8033** | **0.7915** | **0.9845** |

**Supplementary Results & Discussion 1: Blood Flow Gradients**

Blood flow gradients can appear and have been identified by us in multiple scenarios. First, we address flow gradients over a vessel’s cross-section. Larger vessels that have diameters well above the xf-irOCM’s resolution and voxel spacing are known to exhibit laminar flow^1^. In such laminar flows a parabolic flow profile is found (Hagen-Poiseuille equation) with the highest velocity at the center of the vessel and decreasing velocity towards the edges. This parabolic profile is also evident in our measurements. For example, the large vein in Fig. S8a exhibits this and we also add corresponding line profiles of v_z_ as Fig. S1b and S1c from a horizontal line through the vein’s and artery’s center. However, these flow profiles are not explicitly taken into account further but instead are averaged locally as described. In smaller vessels, where the diameter approaches the resolution limit, we are not able to extract flow profiles or equivalently flow gradients over cross-sections anymore. However, when the vessel diameter approaches the size of red blood cells (RBCs), the flow starts deviating from purely laminar flow. In order to deal with sparse single-particle flow in the smallest capillaries, the intralipid injection helps to obtain a stronger signal as the expense of not exactly measuring RBC flow velocity but rather a mixture of RBC and plasma flow velocities.

Flow gradients may also be present along a blood vessel. A vessel segment is always defined as a part of a vessel between two branching points. In such a segment, the pressure drop is constant and flow velocity differences are only caused by a change in diameter along the segment. While in this manuscript we compute an average velocity of each segment for simplicity, the flow along the vessel can be extracted from our data.

**Supplementary Results & Discussion 2: Multiple Scattering Artifacts**

Multiple-scattering artifacts predominantly occur below large pial vessels in both angiograms and Doppler volumes. Because our angiogram segmentation network has been trained to not segment tail artifacts, the artifact afflicted regions underneath large vessels are not considered. Nevertheless, we make some interesting observations in the Doppler power spectrum (DPS) from multiple-scattering affected regions that have implications for multiple scattering below smaller vessels as well. We find that in static tissue below vessels, the DC peak is broadened but no Doppler shift is evident. That is why in Fig. 2c, the axial Doppler signal clearly delineates the vessel while in Fig. 2d, artifacts occur when considering the DPS standard deviation. Furthermore, in the case that vessels with sufficient axial velocity lie inside this region, we have found that the multiple scattering artifacts primarily affect the DC peak, not the shifted Doppler peak. This is also evident Fig. S7b in which the Doppler amplitude angiogram and OCTA angiogram exhibit the well-known tail artifacts. In contrast, the Doppler frequency images (middle) are not afflicted by heavy tail artifacts because the multiple scattering artifacts appear symmetrically around the zero frequency. As a result, capillaries are still overshadowed because the high-pass filter is not able to remove the broadened spectrum around 0 Hz anymore. However, larger penetrating vessels are clearly visible even below major vessels because their respective Doppler shift is strong enough. In general, we find multiple scattering artifacts to be weaker in our data when compared to traditional Gaussian OCM/OCT systems. This may be due to the Bessel beam’s nature to primarily deliver light illumination in the form of a converging ring compared to a strong central Gaussian lobe. In this light, we have also empirically found reduced OCTA tail artifacts in our system compared to Gaussian beam OCTs. Hence, we conclude that our method is sufficiently robust against multiple scattering artifacts, owing to the unique combination of the Bessel-beam illumination, direct assessment of the DPS and advanced segmentation network.
